# Variability in distribution and use of tuberculosis diagnostic tests in Kenya: a cross-sectional survey

**DOI:** 10.1186/s12879-018-3237-z

**Published:** 2018-07-16

**Authors:** J. N. Oliwa, J. Maina, P. Ayieko, D. Gathara, I. A. Kathure, E. Masini, A. H. van’t Hoog, M. B. van Hensbroek, M. English

**Affiliations:** 1 KEMRI-Wellcome Trust Research Programme, Nairobi, Kenya; 2 University of Nairobi, Department of Paediatrics and Child Health, Nairobi, Kenya; 3 National TB and Leprosy Programme, Ministry of Health, Kenya, Nairobi, Kenya; 4 World Health Organisation (Kenya), Nairobi, Kenya; 5 The Academic Medical Centre, University of Amsterdam, Department of Global Health, Amsterdam, The Netherlands; 6 Amsterdam Institute for Global Health and Development, Amsterdam, The Netherlands; 7 Nuffield Department of Medicine, Oxford University, Oxford, UK

**Keywords:** Tuberculosis, Diagnostics, Tests, Distribution, Use, Adults, Children

## Abstract

**Background:**

Globally, 40% of all tuberculosis (TB) cases, 65% paediatric cases and 75% multi-drug resistant TB (MDR-TB) cases are missed due to underreporting and/or under diagnosis. A recent Kenyan TB prevalence survey found that a significant number of TB cases are being missed here. Understanding spatial distribution and patterns of use of TB diagnostic tests as per the guidelines could potentially help improve TB case detection by identifying diagnostic gaps.

**Methods:**

We used 2015 Kenya National TB programme data to map TB case notification rates (CNR) in different counties, linked with their capacity to perform diagnostic tests (chest x-rays, smear microscopy, Xpert MTB/RIF®, culture and line probe assay). We then ran hierarchical regression models for adults and children to specifically establish determinants of use of Xpert® (as per Kenyan guidelines) with county and facility as random effects.

**Results:**

In 2015, 82,313 TB cases were notified and 7.8% were children. The median CNR/100,000 amongst 0-14yr olds was 37.2 (IQR 20.6, 41.0) and 267.4 (IQR 202.6, 338.1) for ≥15yr olds respectively. 4.8% of child TB cases and 12.2% of adult TB cases had an Xpert® test done, with gaps in guideline adherence. There were 2,072 microscopy sites (mean microscopy density 4.46/100,000); 129 Xpert® sites (mean 0.31/100,000); two TB culture laboratories and 304 chest X-ray facilities (mean 0.74/100,000) with variability in spatial distribution across the 47 counties. Retreatment cases (i.e. failures, relapses/recurrences, defaulters) had the highest odds of getting an Xpert® test compared to new/transfer-in patients (AOR 7.81, 95% CI 7.33-8.33). Children had reduced odds of getting an Xpert® (AOR 0.41, CI 0.36-0.47). HIV-positive individuals had nearly twice the odds of getting an Xpert® test (AOR 1.82, CI 1.73-1.92). Private sector and higher-level hospitals had a tendency towards lower odds of use of Xpert®.

**Conclusions:**

We noted under-use and gaps in guideline adherence for Xpert® especially in children. The under-use despite considerable investment undermines cost-effectiveness of Xpert®. Further research is needed to develop strategies enhancing use of diagnostics, including innovations to improve access (e.g. specimen referral) and overcoming local barriers to adoption of guidelines and technologies.

**Electronic supplementary material:**

The online version of this article (10.1186/s12879-018-3237-z) contains supplementary material, which is available to authorized users.

## Background

Globally, there is a significant TB case detection gap: 40% of all tuberculosis (TB) cases, 65% paediatric cases and 75% multi-drug resistant TB (MDR-TB) cases are missed due to a mixture of underreporting and under diagnosis [1–3]. Rapid and accurate diagnosis of TB is critical for timely initiation of treatment to prevent death [4–6]. A recent prevalence survey in Kenya found higher rates of TB than previously thought (558/100,000), with up to 55% of cases being missed probably due to under-detection [7]. Three quarters of the positive TB cases identified in the survey reported seeking care for TB-like symptoms but were not diagnosed. The survey recruited ≥15yr olds, but extrapolation from adult data showed that two thirds of paediatric TB cases were missed [7]. Notification data may underestimate child TB incidence, which may be explained in part by poor reporting of diagnosed paediatric cases [8]; and challenges of diagnosing TB in children due to paucibacillary disease and difficulty obtaining suitable samples [9, 10].

Quicker, more sensitive TB diagnostic technologies are being introduced globally [4, 11]. In 2010, Xpert MTB/RIF® was initially endorsed by the World Health Organisation (WHO) for children, the HIV infected and suspected MDR-TB cases [12], but is now recommended as the first line diagnostic test for all presumed TB cases [13, 14]. Kenya introduced Xpert® in 2011. According to the guidelines used in Kenya in 2015 (when this study was done), all presumptive paediatric, HIV-infected smear negative, drug resistant (DR) cases, or retreatment cases i.e. relapse (recurrence)/defaults/treatment failures should have had at least one Xpert® assay as part of their diagnostic work up (Additional file 1) [15, 16]. By 2015, there were 129 machines distributed throughout the country (146 machines to date), with more expected [17]. Optimal use of Xpert® is important for TB case detection [18, 19]. Few studies have specifically reviewed spatial distribution and practices in the utilisation of bacteriological TB diagnostic tests like Xpert® comparing adults and children, in countries that carry a high TB/MDR burden like Kenya.

A multi-country study on gaps in reporting paediatric TB found children were rarely considered for testing [20]. Age, gender, poverty and literacy are known to influence general health care utilisation and demand for services [21, 22]. One study found socioeconomic status and prior anti-TB treatment were strong determinants of utilisation of bacteriological tests [23]. Behavioural or health system issues may also play a role where services are available but underutilised- perhaps due to patients’ lack of awareness or perceived poor quality [22, 24–26].

Seeking to understand and improve TB case detection in Kenya, we set out to: describe the characteristics and spatial distribution of TB cases reported to the TB programme; the availability, distribution and patterns of use of TB diagnostic tests as per Kenyan guidelines [15, 16]; and to establish the determinants of use of Xpert®, noting differences in adults and children in the various counties. To the best of our knowledge, this is the first attempt to describe in detail the utilisation of TB diagnostics in Kenya, comparing adults and children to try to unmask the “hidden epidemic” of TB [27]. Findings will hopefully help guide policy makers on where the greatest needs are, how well guidelines are being implemented and offer suggestions to mitigate gaps identified. As new tests emerge [28], understanding patterns and determinants of use could help reduce TB deaths by guiding early diagnosis and linkage to appropriate treatment.

## Methods

### Setting

Kenya is administratively divided into 47 counties, that are now largely responsible for health care since devolution in 2013 [29]. Health services are provided by public, private, non-profit non-governmental organisations (NGO) and faith based organisations (FBO). The healthcare system is structured in a hierarchical manner, starting with primary healthcare in the community and complicated cases referred upwards to secondary and tertiary levels of healthcare [29]. According to the Kenyan Master Facility List, there are approximately 10,000 health facilities in the country and just about half are TB treatment sites [30–32]. For this analysis, we aggregated TB health facilities into two: lower level (dispensaries, health centres, and maternity/nursing homes) for primary care; and higher level (county hospitals and national referral facilities) that provide secondary and tertiary referral services.

### Study Population

We included patients of all ages who were notified to the Kenya National TB programme and started TB treatment in 2015.

### Study variables

We wished to explore the possible influence on use of Xpert MTB/RIF® of variables in three hierarchical levels: county, health facility and individual. Individual level co-variates included: age; gender; HIV status; nutritional status; and type of TB patient i.e. new/transfer-in or retreatment cases (relapse/defaults/treatment failures). An age cut-off of 15yrs for children is used locally and internationally for TB programming [14], therefore “child” here refers to 0-14yr olds. For nutrition status, weight-for-age Z (WAZ) scores were computed for those aged 0-23yr and body mass index (BMI) truncated at -5 to +5SD for those ≥10yr, and patients classified as underweight according to the scores. Those 10-23yr who met criteria of underweight by either criterion were also classified as underweight. Health facility level co-variates included: sector of care (public, private, or FBOs), level of care provided (higher vs lower level) and whether they were an Xpert® site or not. County level co-variates included: poverty; maternal education levels; travel time to nearest health facilities; and availability of Xpert® facilities per 100,000. All co-variates were determined for each of the 47 counties for 2015. These factors were decided upon *a priori* following review of literature on drivers of use of TB and health care services in general and data availability [21, 22].

The primary outcome of interest was evidence of Xpert® being done in patients who had been started on TB treatment. According to the 2015 Kenyan guidelines, all presumptive paediatric, HIV-infected smear negative, drug resistant (DR) cases, or retreatment cases i.e. relapse (recurrence)/defaults/treatment failures should have had at least one Xpert® assay as part of their diagnostic work up (Additional file 1) [15, 16].

### Data sources

We considered the best data sources for the three levels of variables of interest. For the individual level, de-identified data from the Kenya National TB Programme patients’ treatment register for 2015 were used (patients who were notified and/or started treatment in 2015). TB case definitions were as per Guidance for National TB programmes (Additional file 1) [33]. Health facility level data were from the Kenya Master Health Facility list [30] and Kenya Services Availability and Readiness Assessment Mapping Report (SARAM) 2014 [34]. Health facilities in the national TB register were geocoded using KEMRI-Wellcome Trust’s Kenya Health Facilities Database, which was last updated in 2016 using online digital place-name gazetteers and Global Positioning System (GPS) sources. County level data were from each county governments’ integrated development plans for 2015 [35]. The projected 2015 Kenya gridded population distribution surface at 100m spatial resolution was obtained from the WorldPop project [36, 37].

### Statistical and Spatial Analysis

Stata version 15MP (StataCorp.2017, College Station, TX, USA) and ArcGIS 10.5 (ESRI, Redlands, CA, USA) were used for statistical analysis, and mapping and spatial analysis respectively. We described the proportion of adults and children reported to the TB programme, their socio-demographic characteristics, use of TB diagnostic tests and outcomes. We used the 2015 Kenya National TB programme data to construct maps for each TB case notification rate (CNR) in different counties and linked this with their capacity to perform diagnostic tests (chest x-rays, smear microscopy, Xpert®, culture and line probe assay) from the SARAM report [34].

For adherence to guidelines, we only had data for those who had tests done, and used these patients to describe patterns of use of TB diagnostic tests. Co-variates of theoretical and/or statistical significance were used to build hierarchical logistic regression models to establish determinants of use of Xpert® in adults and children. Possible collinearity was assessed using the variance inflation factor (VIF). Variables with VIF less than 10 were considered for analysis. The models converged at five integration points for complete case analysis, with county and health facility as random effects [38]. Models were built for the 0-14yrs and ≥15yrs separately and a model for the total population, with likelihood ratio tests, exploration for interactions in pre-specified covariates (HIV and nutrition status) and quantile-quantile plots of residuals used to determine best fit as seen in Additional file 2 [38, 39].

## Results

Data were available from 82,313 patients who started TB treatment in 2015. Table 1 gives the characteristics of these patients for the different age bands and overall population. There were 6,450 children aged 0-14yr, and they represented 7.8% of the total population. In the overall population, 62.4% (51,337) were male, and they were 3,406 (52.8%) in the 0-14yr group. Most of the patients were from the public sector. TB was pulmonary in approximately three quarters of all the patients. A quarter of patients 0-14yrs were HIV infected compared with a third of those ≥15yrs. Overall, close to half the patients were underweight. Close to 10% of those ≥15yrs needed TB retreatment (i.e. failures, relapses/recurrences, defaulters). Less than 5% (309/6,450) of patients 0-14yrs and 12.2% (9,224/75,863) of patients ≥15yrs had an Xpert® done. The proportion of positive Xpert® tests was 63.1% (195/309) for the 0-14yr and 81.2% (7,493/9,224) for the ≥15yrs old; while microscopy was positive in 36.7% (694/1,886) vs 67.1% (40,768/60,797) for 0-14yrs vs the ≥15yrs respectively. Overall case fatality was between 4-6% for both groups.

**Table 1:** Characteristics of Patients notified to the Kenyan National TB Programme in 2015

	0-14yrs n (%)	≥15yrs n (%)	Overall population n (%)
	(N = 6450)	(N = 75863)	(N = 82313)
Gender			
Male	3406 (52.8)	47931 (63.2)	51337 (62.4)
Female	3044 (47.2)	27932 (36.8)	30976 (37.6)
Sector			
Public	4938 (76.6)	59165 (78.0)	64103 (77.9)
Prisons	41 (0.6)	1349 (1.8)	1390 (1.7)
Private	1343 (20.8)	14010 (18.5)	15353 (18.7)
Faith based and others	128 (2.0)	1339 (1.8)	1467 (1.8)
TB Type			
Pulmonary	4774 (74.0)	62964 (83.0)	67738 (82.3)
Extra-pulmonary	1676 (26.0)	12899 (17.0)	14575 (17.7)
HIV testing			
Negative	4517 (70.0)	49016 (64.6)	53533 (65.0)
Positive	1610 (25.0)	24980 (32.9)	26590 (32.3)
Unknown	323 (5.0)	1867 (2.5)	2190 (2.7)
Anthropometry (BMI)			
Underweight^a^	2951 (45.8)	35274 (46.5)	38225 (46.4)
Normal	2777 (43.1)	31338 (41.3)	34115 (41.5)
Overweight/obese	22 (0.34)	2929 (3.9)	2951 (3.4)
Undocumented	700 (10.9)	6322 (8.3)	7022 (8.5)
Type of patient			
New	6233 (96.6)	68470 (90.3)	74703 (90.8)
Transfer in	40 (0.6)	788 (1.0)	828 (1.0)
Relapse (recurrence)	144 (2.2)	5383 (7.1)	5527 (6.7)
Default	33 (0.5)	999 (1.3)	1032 (1.3)
Failure	0 (0)	223 (0.3)	223 (0.3)
Chest-X-ray			
Done	3140 (48.7)	22141 (29.2)	25281 (30.7)
Not done	3310 (51.3)	53722 (70.8)	57032 (69.3)
Smear Microscopy			
Done	1886 (29.2)	60797 (80.1)	62,683 (76.2)
Positive^b^	694 (36.7)	40768 (67.1)	41462 (66.1)
Negative^b^	1192 (63.2)	20029 (32.9)	21221 (33.9)
Xpert MTB/RIF®			
Done	309 (4.8)	9224 (12.2)	9533 (11.6)
Positive (Rif-Sensitive)^b^	195 (63.1)	7493 (81.2)	7688 (80.6)
Positive (Rif-Resistant)^b^	1 (0.3)	129 (1.4)	130 (1.4)
Negative^b^	113 (36.6)	1602 (17.4)	1715 (18.0)
Culture/Line Probe Assay (LPA)			
Done	5 (0.1)	444 (0.6)	449 (0.5)
Drug susceptible^b^	5 (100)	371 (83.6)	376 (83.7)
Drug resistant^b^	0 (0.0)	73 (16.4)	73 (16.3)
Outcome			
Cured	537 (8.3)	31416 (41.4)	31953 (38.8)
Died	270 (4.2)	4391 (5.8)	4661 (5.7)
Treatment failure	1 (0.02)	429 (0.6)	430 (0.5)
Other^c^	5642 (87.5)	39627 (52.2)	45269 (55%)

^a^Underweight defined as either WAZ <-2SD or BMI<18.5 as appropriate for age

^b^Percentage in brackets represent proportions amongst those who got the test done

^c^Other included: treatment not completed; completed but not cured; defaulted; transferred out

### Patterns of use of TB diagnostics tests

**Fig. 1:**
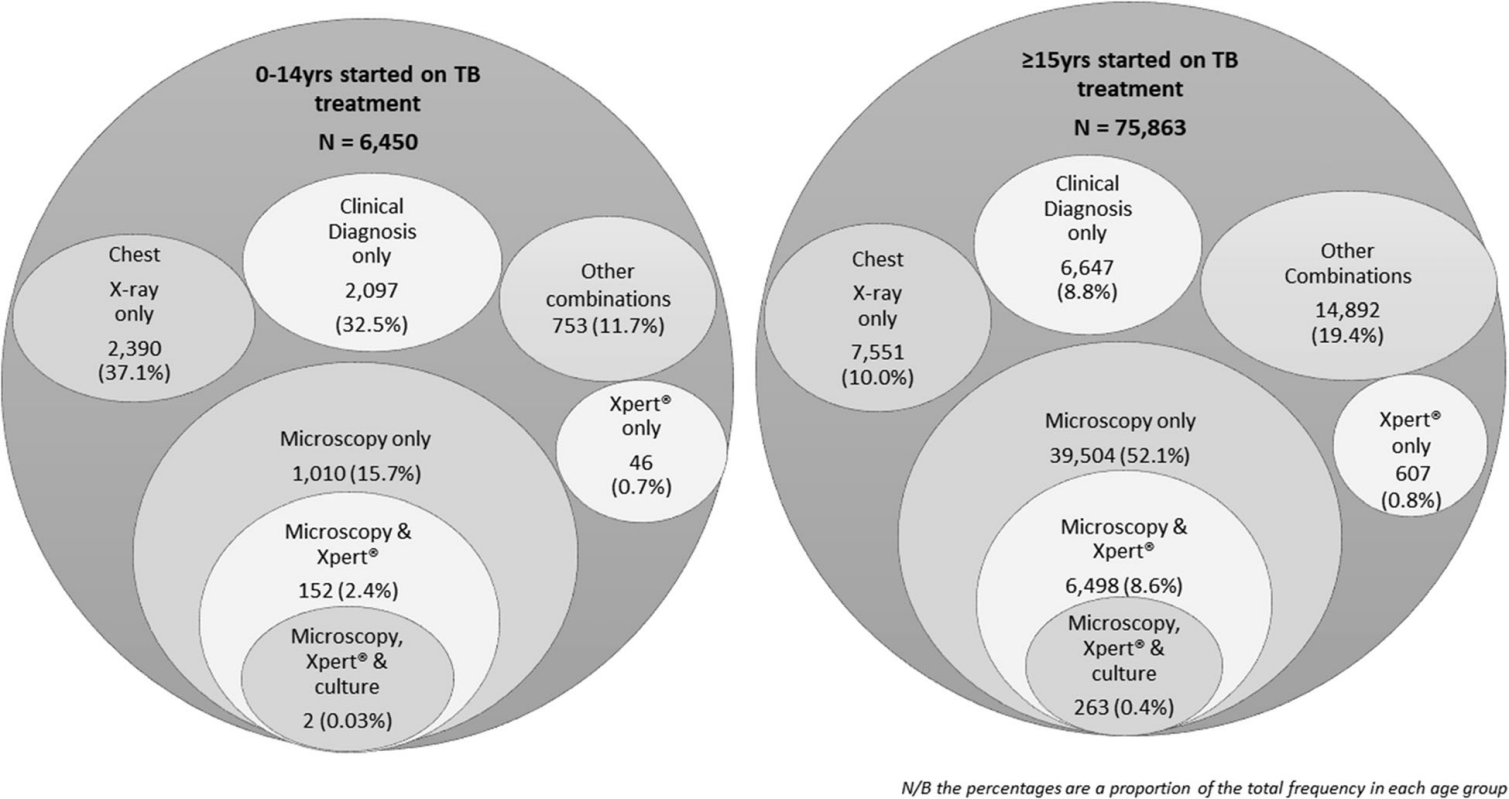
Diagnostic practices amongst patients started on TB treatment in 2015

Figure 1 illustrates diagnostic practices amongst those patients who were started on anti-TB treatment. More than a third of children 0-14yr had no diagnostic test done, and were started on treatment based on clinical diagnosis only. Chest X-ray was the commonly used test in this age group (37.1%); while microscopy was the commonest amongst the ≥15yrs. Table 2 illustrates the patterns of use of bacteriological tests amongst those who had these tests done, relating them to the 2015 Kenyan guidelines (Additional file 1). Children 0-14yr represented <5% of those who had either Xpert® or Microscopy or Culture/LPA done. Those with extra-pulmonary TB also rarely got any of the tests done. Retreatment cases got a culture/LPA done more than any other test. Forty one percent of the patients got Xpert® as per guidelines, with better guideline adherence for culture/LPA (61.2%) and microscopy (97.8%), respectively.

**Table 2:** Patterns of use of TB diagnostics and compared to recommended Kenyan TB guidelines in use in 2015

Characteristic	Xpert® done^a^ *N* = 9,553	Microscopy done^b^ *N*= 62,683	Culture/LPA done^c^ *N* = 515
	n (%)	n (%)	n (%)
Age			
0-14yr	309 (3.2)	1886 (3.0)	5 (1.0)
≥15yr	9224 (96.8)	60797 (97.0)	510 (99.0)
Gender			
Male	6152 (64.5)	40128 (64.0)	385 (74.8)
Type of TB			
Pulmonary	9205 (96.6)	61320 (97.8)	508 (98.6)
Extrapulmonary	328 (3.4)	1363 (2.2)	7 (1.4)
Type of patient			
New/transfer in	6774 (71.1)	56821 (90.7)	200 (38.8)
Retreatment (relapse/defaults/treatment failures)	2759 (28.9)	5862 (9.3)	315 (61.2)
Test done as per guideline	3909^a^ (41.0)	61320^b^ (97.8)	315^c^ (61.2)

Excerpt from 2015 Kenyan guidelines

^a^Xpert® recommended for all presumptive paediatric TB OR HIV-infected smear negative OR drug resistant cases OR retreatment cases i.e. relapse/defaults/treatment failures in adults OR suspected drug resistant TB

^b^Microscopy recommeded for all recommended pulmonary tuberculosis cases

^c^Culture/LPA recommended for retreatment cases ((i.e. relapse/defaults/treatment failures) regardless of age

### Tuberculosis Case Notification Rates and distribution of TB cases in Kenya

**Fig. 2:**
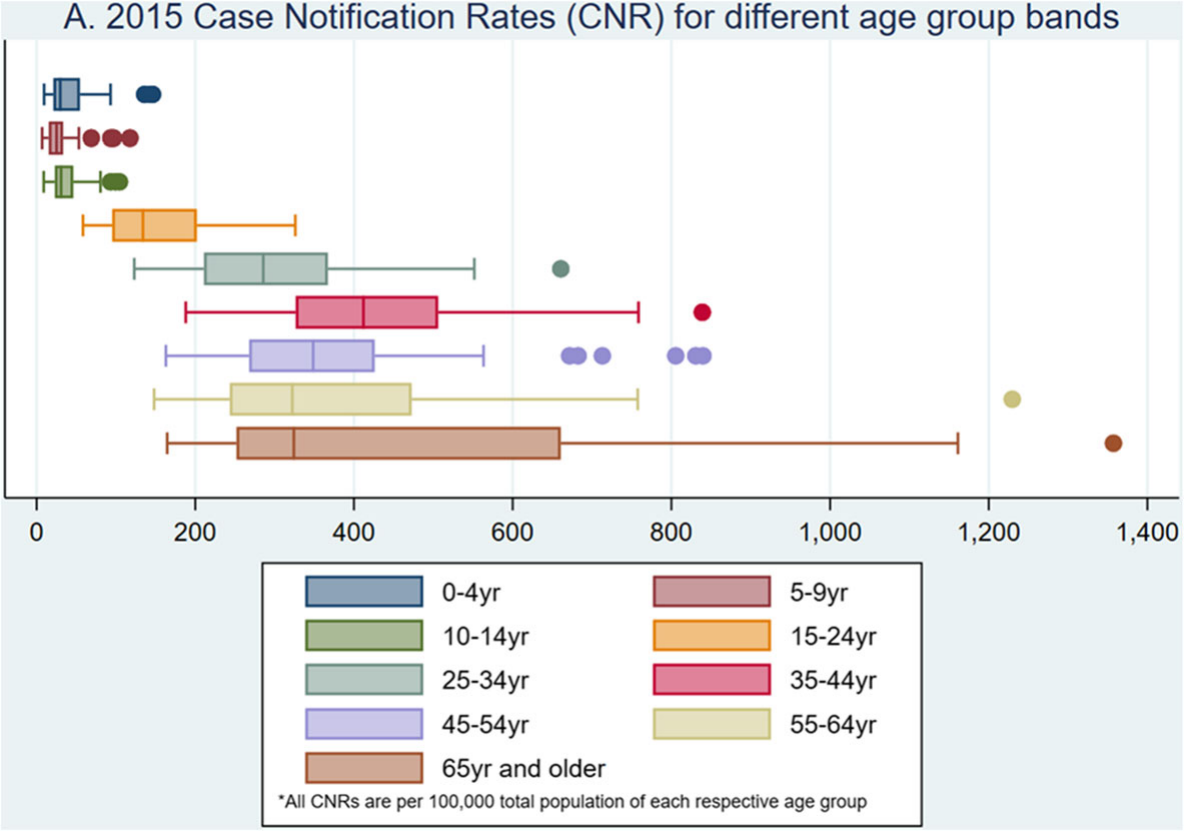
TB Case Notification Rates (CNRs) for different age group bands in Kenya

**Fig. 3:**
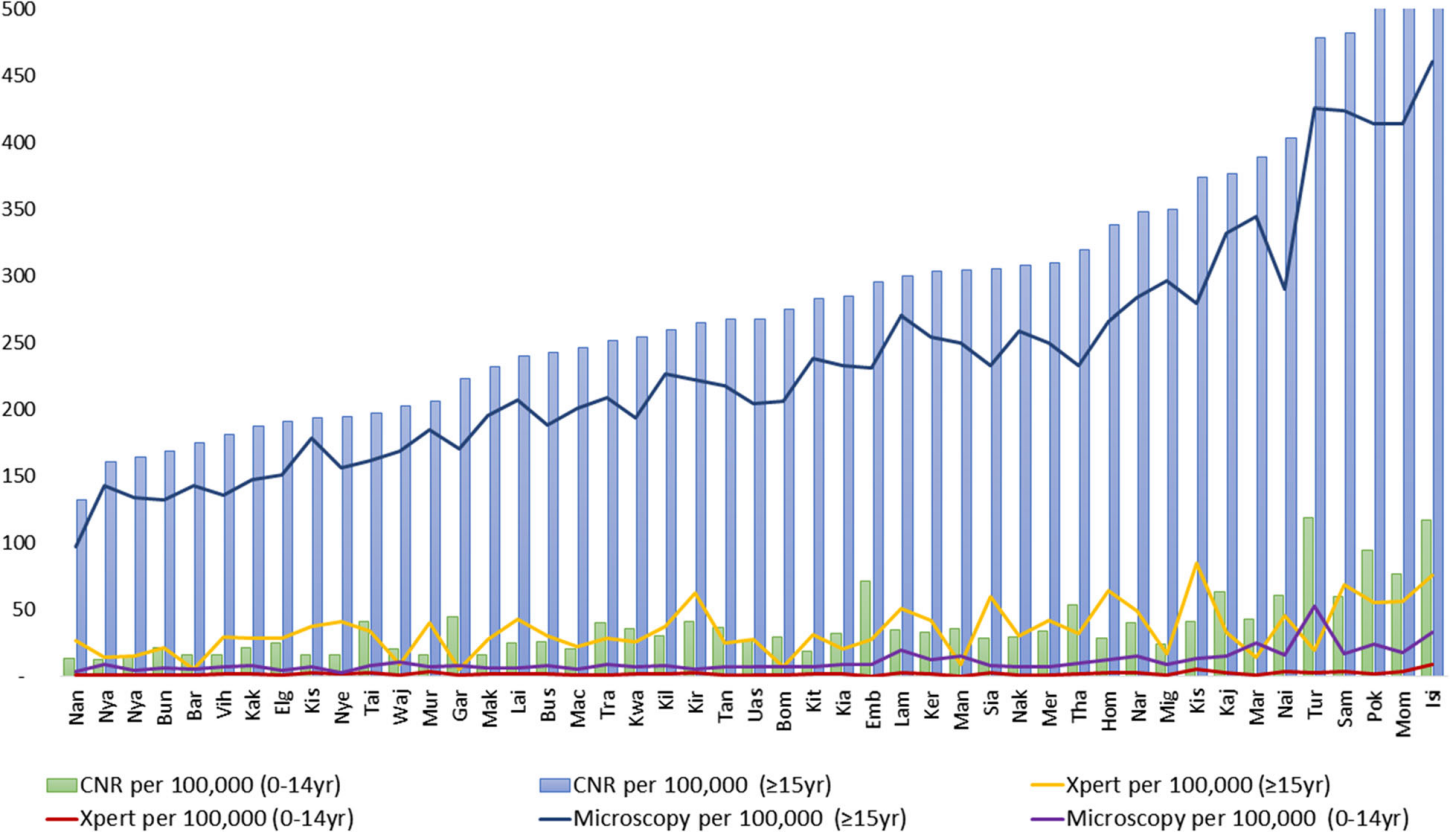
Chart showing variability of County TB CNRs and use of Xpert® and Microscopy per 100,000 population comparing adults and children (Full county names and variables in Additional file 4)

Figures 2 and 3 and Additional files 3 and 4 show case notification rates (CNR) by age group and county, as well as variation in county use of Xpert® and microscopy. Median age was 32yr (IQR 24yr, 43yr). The highest CNR was amongst those 35-44yrs (455/100,000), and the lowest amongst the 5-9yrs (27/100,000) Fig. 2. The median CNR/100,000 amongst children 0-14yr old was 37.2 (IQR 20.6, 41.0) and 267.4 (IQR 202.6, 338.1) for ≥15yr olds respectively. Median use of Xpert® among 0-14yr olds was 1.45/100,000 (IQR 0.7, 2.1) and in ≥15yr olds was 29.9/100,000 (IQR 21.0, 42.8). Median use of microscopy among 0-14yr olds was 8.1/100,000 (IQR 6.6, 12.8) and 222/100,000 (IQR 169.8, 270.4) in ≥15yr olds. Xpert® use did not correlate with increase in county TB CNR/100,000 (Fig. 3 and Additional file 4) and in this was also observed in the univariate analysis where counties with higher Xpert® density did not have significantly higher CNRs (Additional file 2). Microscopy use in the ≥15yr tended to be higher in counties with highest CNRs (Fig. 3 and Additional file 4).

**Fig 4:**
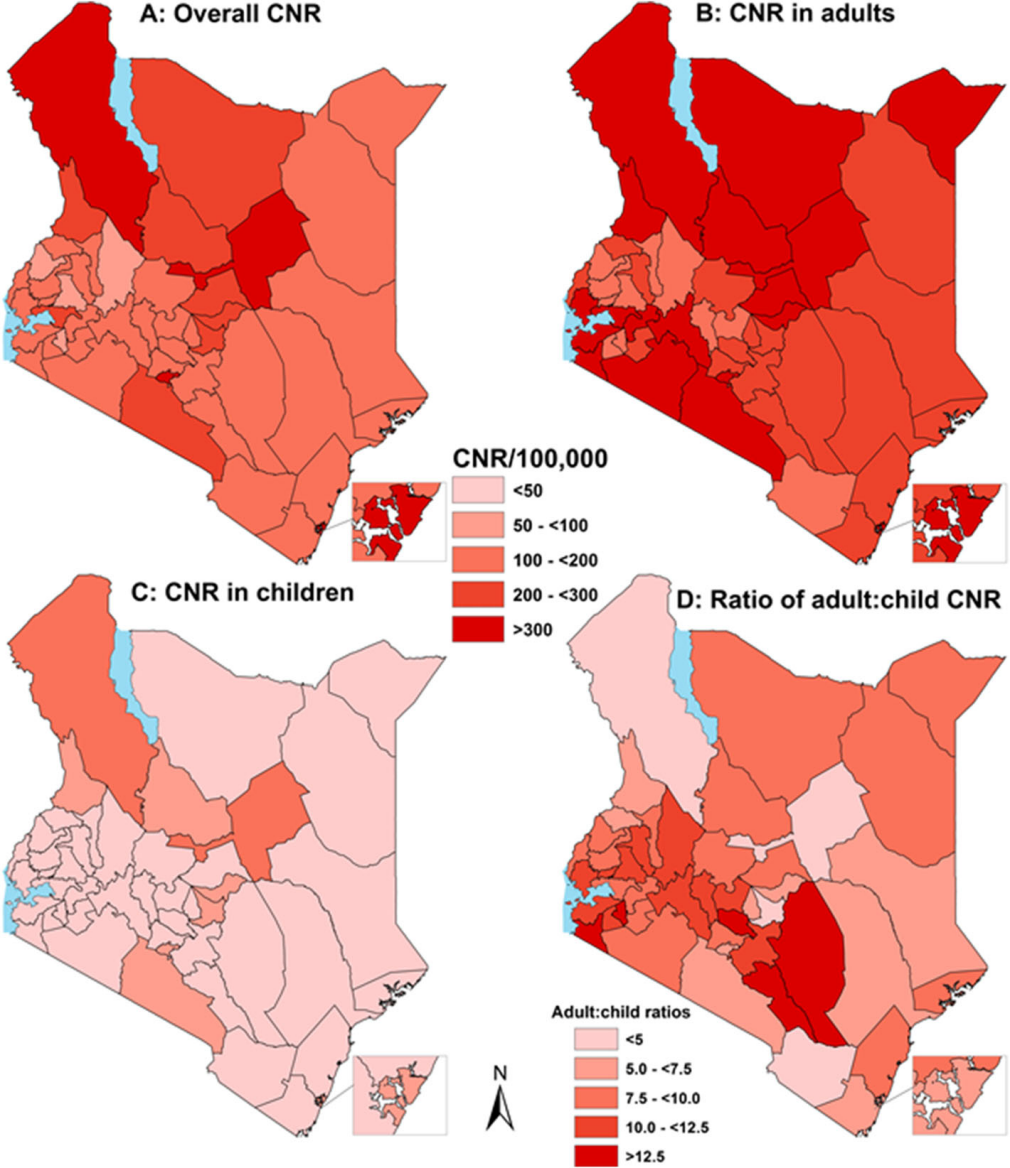
Maps illustrating the spatial distribution of TB patients in various counties in Kenya by: Overall population CNR/100,000 (**a**); CNR/100,000 in “adults” i.e. ≥15yrs (**b**); CNR/100,000 in children 0-14yrs (**c**); and ratio of adult: child TB CNRs (**d**)

Figure 4 shows a panel of maps illustrating spatial distribution of TB patients in Kenya. The counties with two large cities had some of the highest CNRs, as did two counties serving the pastoralist communities of the northern and eastern frontiers (panel A). Panel B and C show the CNRs in the ≥15yr olds (“Adults”) and in children 0-14yr old respectively, while D shows the ratio of adult to child TB cases. These maps highlight some of the adult hot spots with low child CNRs and ensuing differences in adult: child ratios highlighting the gaps in case detection for children.

**Fig. 5:**
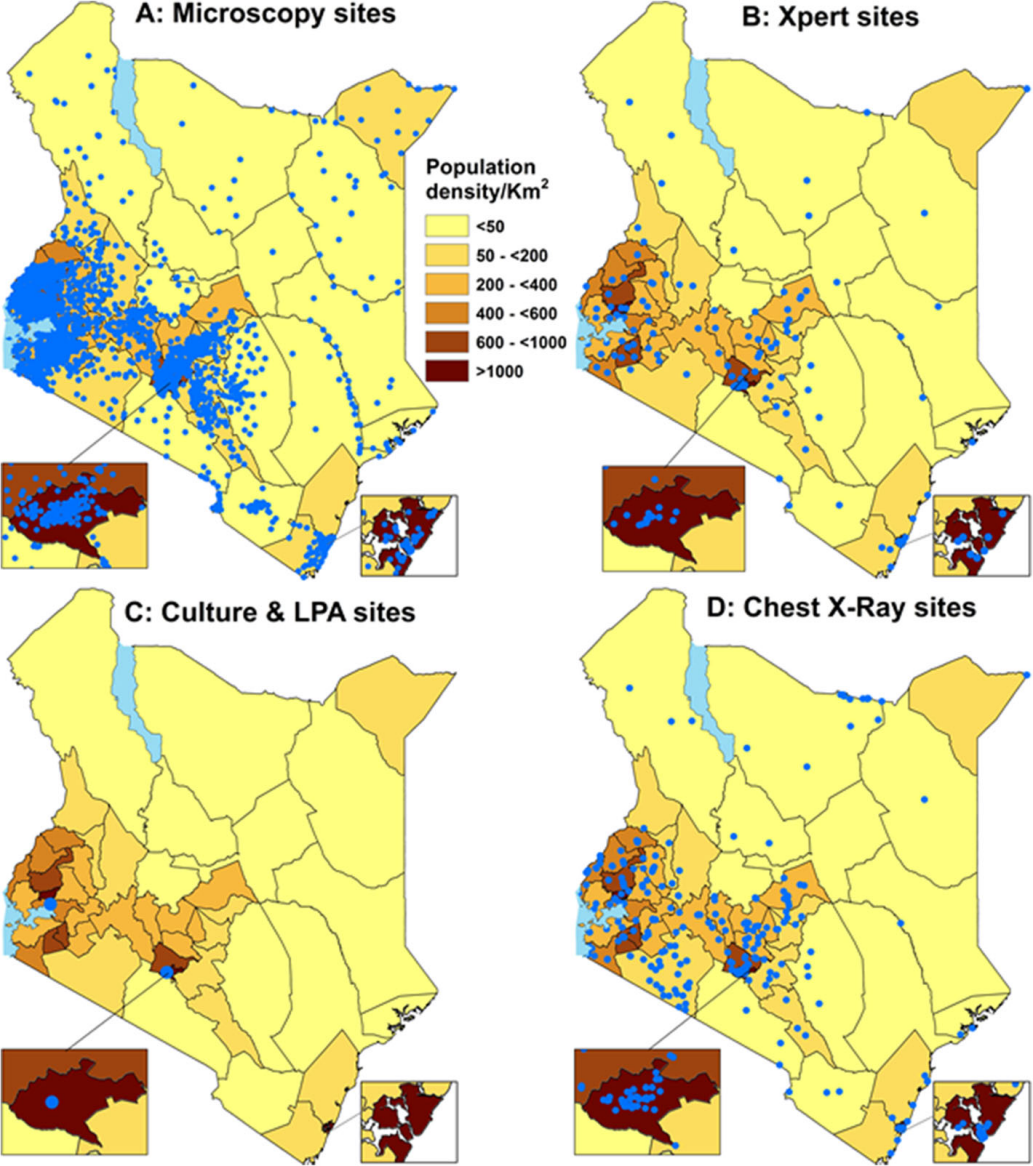
Maps illustrating the distribution of facilities with TB diagnostic capabilities by: Microscopy (**a**); Xpert MTB/RIF® (**b**); Culture and Line Probe Assay Labs (**c**); and Chest X-ray (**d**), all overlaying each county’s population density in 2015 as the background

Figure 5 and Additional file 5 show the distribution of facilities with TB diagnostic facilities (Microscopy-Map A; Xpert MTB/RIF®-Map B; Culture and Line Probe Assay- Map C; and chest X Ray-Map D) overlaid with each county’s total population density. In 2015, there were 129 facilities providing Xpert® services (mean Xpert® density of 0.28/100,000); 304 chest X ray facilities (mean density 0.67/100,000); and 2,072 facilities with microscopy services (mean density 4.54/100,000). There were disparities noted in the distribution of Xpert® (panel B) and chest X-ray (panel D) facilities with some northern and eastern counties of Kenya having low facility densities/100,000 yet they carry a high burden of TB cases (Fig. 4).

### Determinants of use bacteriological TB diagnostic tests comparing adults and children

Additional file 2 shows the univariate analysis of the factors that had been considered for the model building, and Table 3 shows the final adjusted hierarchical models of determinants of use of Xpert® in adults and children, with county and health facility as random effects. Amongst individual level factors, the retreatment cases (i.e. failures, relapses/recurrences, defaulters) had the highest odds of getting an Xpert® (total population AOR 7.81, 95% CI 7.33 to 8.33). From the model of the total population, children had reduced odds of getting an Xpert® test (AOR 0.41, CI 0.36 to 0.47), while being male was associated with increased odds in all age groups. Overall, the HIV positive individuals had nearly twice the odds of getting an Xpert® compared to the HIV negative. Nutrition status had a marginal effect, except in the 0-14yr olds where the underweight group had nearly twice the odds of getting an Xpert® compared with the well-nourished cases.

**Table 3:** Determinants of use Xpert/MTB/RIF® in Kenya

Characteristic	0-14yrs Xpert® done n (column %) *N*=309	≥15yrs Xpert® done n (column %) *N*=9,224	Model 1 0-14yrs adjusted OR (95% CI) *N*=6,450	Model 2 ≥15yrs adjusted OR (95% CI) *N*=75,845	Model 3 Overall adjusted OR (95% CI) *N*=82,295
Age					1 (≥15yrs base) 0.41 (0.36 to 0.47)
Gender					
Female	140 (45.3)	3241 (35.1)	1 (base)	1 (base)	1 (base)
Male	169 (54.7)	5983 (64.9)	1.13 (0.88 to 1.45)	1.09 (1.03 to 1.14)	1.09 (1.03 to 1.14)
HIV Status					
Negative	164 (53.1)	4751 (51.5)	1 (base)	1 (base)	1 (base)
Positive	143 (46.3)	4372 (47.4)	2.00 (1.52 to 2.61)	1.83 (1.74 to 1.93)	1.82 (1.73 to 1.92)
Unknown	2 (0.7)	101 (1.1)	0.19 (0.05 to 0.78)	0.81 (0.65 to 1.01)	0.76 (0.61 to 0.94)
Nutrition Status					
Normal	40 (12.9)	3379 (36.6)	1 (base)	1 (base)	1 (base)
Underweight	238 (77.0)	5132 (55.6)	1.97 (1.36 to 2.83)	1.13 (1.07 to 1.19)	1.15 (1.09 to 1.21)
Overweight/obese	3 (1.0)	360 (3.9)	0.94 (0.26 to 3.36)	0.88 (0.77 to 0.99)	0.88 (0.78 to 1.00)
Unknown	28 (9.1)	353 (3.8)	1.34 (0.79 to 2.27)	0.64 (0.56 to 0.73)	0.67 (0.59 to 0.76)
Type of patient					
New patient/transfer in	277 (89.6)	6497 (70.4)	1 (base)	1 (base)	1 (base)
Relapse/Default/Failure	32 (10.4)	2727 (29.6)	4.19 (2.60 to 6.75)	7.97 (7.47 to 8.50)	7.81 (7.33 to 8.33)
Sector					
Public	242 (78.3)	7692 (83.4)	1 (base)	1 (base)	1 (base)
Private	62 (20.1)	1373 (14.9)	0.93 (0.63 to 1.37)	0.85 (0.75 to 0.97)	0.85 (0.75 to 0.97)
FBOs^a^	5 (1.6)	159 (1.7)	0.62 (0.19 to 2.11)	0.99 (0.67 to 1.46)	0.99 (0.68 to 1.45)
Health Facility Level					
Lower level^b^	174 (56.3)	5737 (62.2)	1 (base)	1 (base)	1 (base)
Higher level^c^	120 (38.8)	3165 (34.3)	1.18 (0.80 to 1.74)	0.91 (0.79 to 1.05)	0.91 (0.79 to 1.06)
Unknown	15 (4.9)	322 (3.5)	1.99 (1.01 to 3.92)	1.01 (0.82 to 1.26)	1.04 (0.84 to 1.28)
Patient from an Xpert® site					
Not from Xpert® site	228 (73.8)	6778 (73.5)	1 (base)	1 (base)	1 (base)
From Xpert® site	81 (26.2)	2446 (26.5)	1.17 (0.74 to 1.85)	2.30 (1.86 to 2.82)	2.23 (1.82 to 2.74)
County Poverty Levels (from poorest to richest)					
Quartile 1	53 (17.2)	1839 (19.9)	1 (base)	1 (base)	1 (base)
Quartile 2	78 (25.2)	2875 (31.2)	0.85 (0.39 to 1.86)	1.22 (0.60 to 2.48)	1.19 (0.60 to 2.37)
Quartile 3	103 (33.3)	2802 (30.4)	1.04 (0.48 to 2.26)	1.34 (0.66 to 2.74)	1.32 (0.66 to 2.62)
Quartile 4	75 (24.3)	1708 (18.5)	0.54 (0.25 to 1.18)	0.64 (0.32 to 1.27)	0.62 (0.32 to 1.21)
Intra-class correlation coefficients (ICC)					
County			0.04 (0.02 to 0.11)	0.07 (0.04 to 0.11)	0.07 (0.04 to 0.11)
Health facility			0.26 (0.18 to 0.37)	0.24 (0.21 to 0.27)	0.24 (0.21 to 0.27)

^a^*FBOs* Faith Based Organisations

^b^Primary referral facilities

^c^Secondary and tertiary referral facilities

Examining factors at the facility and county levels, patients in private sector and from higher level facilities had a tendency towards lower odds of getting tested compared with those in public sector or lower level facilities, but this difference was not statistically significant for the 0-14yrs old. We also noted that counties with higher Xpert® density did not report significantly higher CNRs. From the intra-class correlation coefficients (ICC), county as a level explained approximately 4 & 7% and health facility 24 & 26% of the observed variability in each adjusted model.

## Discussion

We set out to describe the spatial distribution of TB patients and TB diagnostic services, and patterns of use of TB diagnostic tests in Kenya as per the guidelines (with emphasis on Xpert MTB/RIF®), noting differences in children and adults. The TB case notification rate (CNR) in children 0-14yrs was nearly eight times less than that of those ≥15yrs, which may imply under detection. Children are thought to represent 10-20% of the total reported TB cases in high TB endemic settings like Kenya, but we observed only 7.8% in our data [40, 41]. Low CNR among children could additionally be explained by underreporting due to difficulties in confirming a diagnosis of TB in them and poor surveillance [2].

We observed wide county variation in distribution and use of TB diagnostic tests, as well as in case notification rates. Some of the northern and eastern counties had high TB CNRs, but a lower density/100,000 of facilities equipped with TB diagnostic services. Conversely, some counties had higher facility densities but reported lower rates of use. Kenya’s health system has been affected by devolution and decentralisation of health services, and this could be having a bearing on TB care [42]. A recently published patient pathway analysis in Kenya found distinct variation in diagnostic and treatment availability across counties and facility levels [32].

To comply with existing guidelines at the time, all presumed child TB cases, HIV infected adults and suspected MDR-TB cases and retreatment cases should have had at least an Xpert® done as part of the diagnostic work up [15, 16]. We noted underutilisation, with less than 5% of patients 0-14yrs and 12.2% of patients ≥15yrs having an Xpert MTB/RIF® test done, with gaps in guideline adherence. Many children, however, had smear microscopy done, on a specimen that presumably could have been used for Xpert®. The assay has much better sensitivity than smear microscopy and can identify rifampicin resistance with much faster turn-around time than traditional culture, and has been shown to be cost effective [43–46]. The Kenya prevalence survey found that when microscopy was used alone, up to 50% of TB cases were being missed, while Xpert MTB/RIF® detected 78% of TB cases [7]. While use of Xpert® is encouraged as the more sensitive test, it still misses approximately 22% of TB cases, and a negative Xpert®, especially in children does not rule out TB [43, 44]. However, if health workers can get sputum for microscopy especially in children, then they should ideally be able to send the sample for Xpert®, the recommended first-line test.

From the regression analysis, retreatment cases (i.e. failures, relapses/recurrences, defaulters) had the greatest odds of getting an Xpert® as expected from the guidelines, as did those who were HIV infected after adjusting for other covariates. Children had reduced odds of getting tested, despite the recommendation that all should have had an Xpert®. There is, however, no documentation of failed attempts to test.

Low utilisation of Xpert® has been illustrated in other low income, high TB burden countries both in adults and children [47–49]. From surveys of implementation in high TB burden countries including Kenya, reasons associated with low utilisation included operational issues like power outages and poor specimen referral; doubts about impact to TB morbidity and mortality; preference to trust clinical acumen; low sensitivity especially in children; challenges in getting good specimens and false negatives; and lack of awareness amongst health care workers and patients [45, 48, 50]. In Kenya, Xpert® sensitisation was initially done for lab staff only, which could have led to less demand for the test from clinicians. Some studies identified that training, workload, administrative support, staff motivation, role models and participation in the guideline development influence the implementation of TB guidelines in general [51–53].

Patients from higher level facilities had a tendency towards reduced odds of getting an Xpert® done. This could be because they get patients already being worked up from lower level facilities being referred to them to manage complications. The Kenya TB patient pathway analysis paper found that 58% of patients sought care in lower level facilities [32]. The Kenya SARAM report found only 60% of Kenya’s health facilities were ready to provide Kenya Essential Package for Health-defined TB services [34]. Readiness was found to be highest at the primary care facilities and in public facilities [34]. This is unlike in more well-resourced low TB burden countries, where TB care is at tertiary level facilities, and most patients have access to molecular diagnostic tests [54]. Private sector patients also had a tendency toward reduced odds of getting tested compared to public sector patients. This could be explained by private practice patients having to pay for tests to be done, and physicians may fail to comply with national guidelines, as seen in other settings [55–57].

### Limitations

These data are from notification data which may be incomplete because an unknown number of cases may be treated but not reported. We additionally had no information on those in whom a test was done and not documented, or in whom a test was attempted but failed. A large proportion of relevant cases probably had no record of a test being done and no indication of why a test was not done.

This work, however, still provides much needed insights about the utilisation of diagnostic tests amongst those accessing health care who have been diagnosed and started on TB treatment. It also contrasts with the prevalence survey, where up to 40% of TB cases were missed and, thus, got no TB diagnostic tests. The guidelines recommend that all patients should have a bacteriological test prior to treatment. We evaluated, with the information available, how well the guidelines were being followed.

Our complete case analysis dropped approximately 3% of the cases due to missing data on facility, but this did not reduce our ability to make meaningful inferences. We also considered all the 0-14yrs old as a single group but there may be differences in the sub categories. Because the numbers of children were low, subgroup analysis could have been misleading. This analysis together with the prevalence survey still provide deep insights into the known TB cases and guideline adherence, which can aid in planning services to improve TB case detection, especially in children.

## Conclusions

Underuse of Xpert® despite wide scale roll out and considerable investments undermines assertions of the cost-effectiveness of this technology. There is need to increase use of Xpert®, to ensure the correct patients are targeted for lengthy and potentially toxic TB treatment, and so that its potential advantages are realised and investments justified. Current focus on increasing access to Xpert® is insufficient and more attention needs to be given to service delivery models that involve and educate staff and that address potential challenges of patient and specimen referral, with consideration needed for the private sector. Further research can support such efforts by identifying barriers and testing strategies to overcome these.

## Additional files

Additional file 1: Guidelines and TB Case Definitions. (DOCX 65 kb)

Additional file 2: Univariate analysis and Model Diagnostics. (DOCX 96 kb)

Additional file 3: Distribution of TB cases by age group for the various counties. (DOCX 27 kb)

Additional file 4: TB Diagnostic tests by county per 100,000 total population. (DOCX 23 kb)

Additional file 5: County Names, Case Notification Rates and use of Xpert® and Microscopy. (DOCX 21 kb)

## Abbreviations

AOR: Adjusted Odds Ratios
BMI: Body Mass Index
CNR: Case Notification rate
DR: Drug-resistant
FBO: Faith-Based Organisations
HIV: Human Immune-deficiency Virus
IQR: Inter Quartile Ratio
LPA: Line Probe Assay
MDR-TB: Multidrug resistance Tuberculosis
NGO: Non-Governmental Organisations
SARAM: Services Availability and Readiness Assessment Mapping Report
TB: Tuberculosis
VIF: Variance Inflation Factor
WAZ: Weight-for-Age-Z score
WHO: World Health Organisation
Xpert®: Xpert MTB/RIF®
