# Supplementary material for: Variability in distribution and use of tuberculosis diagnostic tests in Kenya: a cross-sectional survey
Source: BMC Infect Dis. 2018 Jul 16;18:328. doi: 10.1186/s12879-018-3237-z (PMC6048895; doi:10.1186/s12879-018-3237-z)
Supplement: Supplementary file 1 — Guidelines and TB Case Definitions. (DOCX 65 kb) [file 12879_2018_3237_MOESM1_ESM.docx]

## Additional File 1: Guidelines and TB Case Definitions

Kenya Adult and Paediatric TB diagnostic algorithms in use for presumptive TB patients as per 2015 [26, 27]


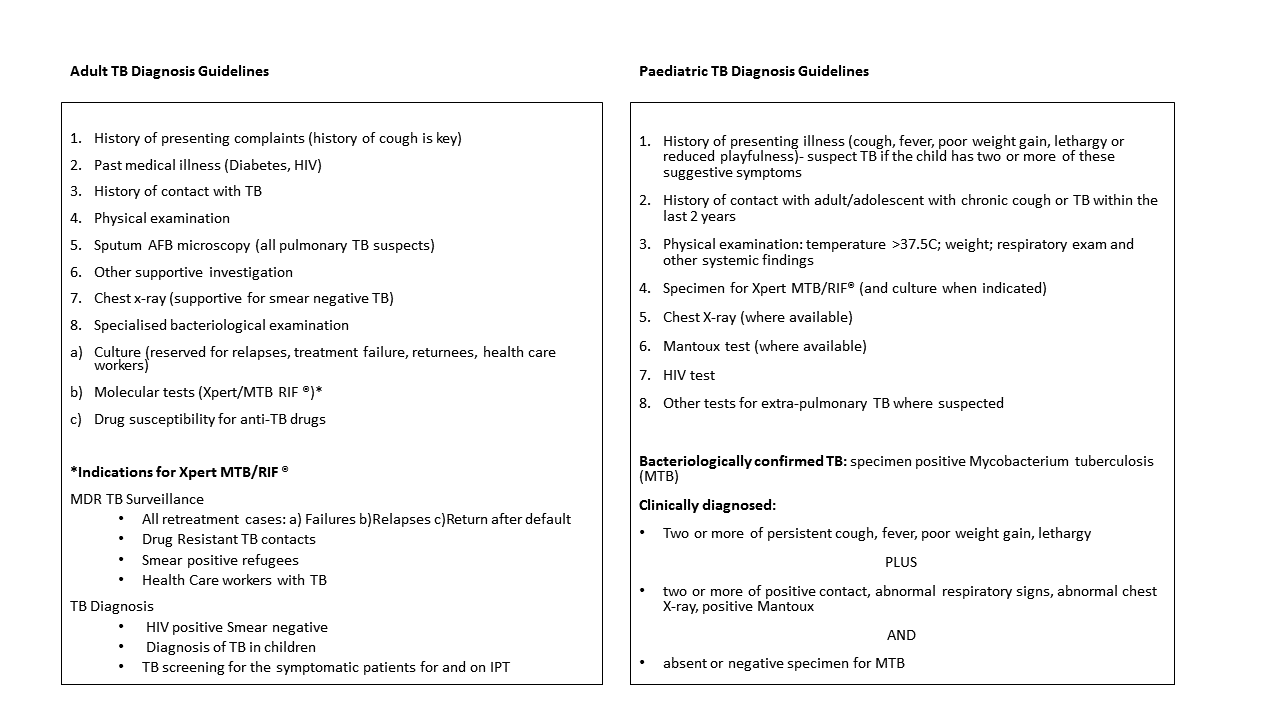


**TB Case definitions and Treatment outcomes for Drug susceptible TB patients [34]**

| **Outcome** | **Definition** |
| --- | --- |
| Cured | A pulmonary TB patient with **bacteriologically confirmed*** TB at the beginning of treatment who was smear- or culture-negative in the last month of treatment and on at least one previous occasion  * A *bacteriologically confirmed TB case* is one from whom a biological specimen is positive by smear microscopy, culture or WHO-approved rapid diagnostics (such as Xpert MTB/RIF).  * A *clinically diagnosed TB case* is one who does not fulfil the criteria for bacteriological confirmation but who has been diagnosed with active TB by a clinician or other medical practitioner who has decided to give the patient a full course of TB treatment. This definition includes cases diagnosed on the basis of X-ray abnormalities or suggestive histology and extra-pulmonary cases without laboratory confirmation. Clinically diagnosed cases subsequently found to be bacteriologically positive (before or after starting treatment) should be reclassified as bacteriologically confirmed  * *Presumptive TB* refers to a patient who presents with symptoms or signs suggestive of TB (previously known as a TB suspect) |
| Treatment completed | A TB patient who completed treatment without evidence of failure *but* with no record to show that sputum smear or culture results in the last month of treatment and on at least one previous occasion were negative, either because tests were not done or because results are unavailable |
| Treatment failed | A TB patient whose sputum smear or culture is positive at month 5 or later during treatment |
| Died | A TB patient who dies for any reason before starting or during the course of treatment |
| Lost to follow-up | A TB patient who did not start treatment or whose treatment was interrupted for 2 consecutive months or more |
| Not evaluated | A TB patient for whom no treatment outcome is assigned. This includes cases “transferred out” to another treatment unit as well as cases for whom the treatment outcome is unknown to the reporting unit. |
| Treatment success | The sum of *cured* and *treatment completed* |
