# Supplementary material for: Variability in distribution and use of tuberculosis diagnostic tests in Kenya: a cross-sectional survey
Source: BMC Infect Dis. 2018 Jul 16;18:328. doi: 10.1186/s12879-018-3237-z (PMC6048895; doi:10.1186/s12879-018-3237-z)
Supplement: Supplementary file 2 — Univariate analysis and Model Diagnostics. (DOCX 96 kb) [file 12879_2018_3237_MOESM2_ESM.docx]

## Additional File 5: Univariate analysis and Model Diagnostics

| **Characteristic** | **0-14yrs**  **Xpert done**  **n (%*)**  **N=309** | **≥15yrs**  **Xpert done**  **n (%*)**  **N=9,224** | **0-14yrs**  **unadjusted OR**  **(95% CI)**  N= | **≥15yrs**  **unadjusted OR**  **(95% CI)**  N= | **Overall**  **unadjusted OR**  **(95% CI)**  **N= 82,295** |
| --- | --- | --- | --- | --- | --- |
| **Age** | | | | | |
| *0-14yr (n=6,450)* | 309 (4.8) |  | 0.34 (0.30 to 0.38) |  |  |
| *≥15yrs (n=75,863)* |  | 9,224 (12.2) | 1 (base) | 2.95 (2.61 to 3.33) |  |
| **Gender** | | | | | |
| *Female* | 140 (45.3) | 3,241 (35.1) | 1 (base) | 1 (base) | 1 (base) |
| *Male* | 169 (54.7) | 5,983 (64.9) | 1.14 (0.89 to 1.45) | 1.09 (1.03 to 1.14) | 1.11 (1.06 to 1.17) |
| **HIV Status** | | | | |  |
| *Negative* | 164 (53.1) | 4,751 (51.5) | 1 (base) | 1 (base) | 1 (base) |
| *Positive* | 143 (46.3) | 4,372 (47.4) | 2.36 (1.82 to 3.07) | 1.89 (1.80 to 1.99) | 1.93 (1.83 to 2.02) |
| *Unknown* | 2 (0.7) | 101 (1.1) | 0.18 (0.04 to 0.75) | 0.72 (0.58 to 0.89) | 0.66 (0.53 to 0.81) |
| **Nutrition Status** | | | | |  |
| *Normal* | 40 (12.9) | 3,379 (36.6) | 1 (base) | 1 (base) | 1 (base) |
| *Underweight* | 238 (77.0) | 5,132 (55.6) | 2.20 (1.54 to 3.16) | 1.20 (1.14 to 1.26) | 1.17 (1.11 to 1.23) |
| *Overweight/obese* | 3 (1.0) | 360 (3.9) | 1.06 (0.30 to 3.68) | 0.79 (0.70 to 0.89) | 0.82 (0.72 to 0.92) |
| *Unknown* | 28 (9.1) | 353 (3.8) | 1.45 (0.86 to 2.44) | 0.68 (0.60 to 0.77) | 0.66 (0.58 to 0.74) |
| **Type of patient** | | | | | |
| *New/Transfer in* | 277 (89.6) | 6,497 (70.4) | 1 (base) | 1 (base) | 1 (base) |
| *Failure/Relapse/Default* | 32 (10.4) | 2,727 (29.6) | 5.37 (3.36 to 8.59) | 8.31 (7.79 to 8.86) | 8.44 (7.92 to 8.99) |
| **Sector** | | | | | |
| *Public & Prisons* | 242 (78.3) | 7,692 (83.4) | 1 (base) | 1 (base) | 1 (base) |
| *Private* | 62 (20.1) | 1,373 (14.9) | 0.99 (0.68 to 1.45) | 0.76 (0.67 to 0.87) | 0.77 (0.69 to 0.87) |
| *FBOs** | 5 (1.6) | 159 (1.7) | 0.71 (0.21 to 2.34) | 0.94 (0.64 to 1.37) | 0.93 (0.64 to 1.37) |
| **County Poverty Levels (from poorest to richest)** | | | | | |
| *Quartile 1* | 53 (17.2) | 1,839 (19.9) | 1 (base) | 1 (base) | 1 (base) |
| *Quartile 2* | 78 (25.2) | 2,875 (31.2) | 1.16 (0.61 to 2.18) | 1.18 (0.57 to 2.43) | 1.15 (0.56 to 2.37) |
| *Quartile 3* | 103 (33.3) | 2,802 (30.4) | 1.52 (0.81 to 2.86) | 1.23 (0.60 to 2.53) | 1.19 (0.58 to 2.46) |
| *Quartile 4* | 75 (24.3) | 1,708 (18.5) | 1.38 (0.75 to 2.53) | 0.56 (0.28 to 1.12) | 0.53 (0.26 to 1.06 |
| **Maternal Education** | | | | | |
| *Low levels* | 233 (75.4) | 7,270 (78.8) | 1 (base) | 1 (base) | 1 (base) |
| *Moderate/high* | 76 (24.6) | 1,954 (21.2) | 1.09 (0.79 to 1.52) | 1.32 (1.26 to 1.37) | 0.54 (0.36 to 0.79) |
| **Time to travel to health facilities** | | | | | |
| *Short travel* | 148 (47.9) | 4,217 (45.7) | 1 (base) | 1 (base) | 1 (base) |
| *Medium travel* | 81 (26.2) | 2,937 (31.8) | 0.76 (0.52 to 1.11) | 1.35 (1.30 to 1.41) | 0.86 (0.52 to 1.42) |
| *Long travel* | 80 (25.9) | 2,070 (22.4) | 0.80 (0.54 to 1.17) | 1.44 (1.38 to 1.51) | 0.57 (0.35 to 0.94) |
| **Health Facility Level** | | | | | |
| *Lower level*** | 174 (56.3) | 5,737 (62.2) | 1(base) | 1(base) | 1 (base) |
| *Higher level**** | 120 (38.8) | 3,165 (34.3) | 1.22 (0.88 to 1.70) | 1.19 (1.05 to 1.35) | 1.17 (1.04 to 1.33) |
| *Unknown* | 15 (4.9) | 322 (3.5) | 1.78 (0.91 to 3.49) | 1.02 (0.82 to 1.26) | 1.03 (0.84 to 1.27) |
| **Patient from an Xpert site** | | | | | |
| *Not from site* | 228 (73.8) | 6,778 (73.5) | 1(base) | 1(base) | 1 (base) |
| *From site* | 81 (26.2) | 2,446 (26.5) | 1.25 (0.85 to 1.85) | 1.72 (1.64 to 1.81) | 2.11 (1.77 to 2.53) |
| **Facility density of Xpert testing facilities/100,000** | | | | |  |
| *Low* | 115 (37.2) | 3,186 (34.5) | 1 (base) | 1 (base) | 1 (base) |
| *Moderate* | 62 (20.1) | 2,447 (26.5) | 1.15 (0.81 to 1.63) | 1.26 (1.21 to 1.31) | 0.78 (0.48 to 1.28) |
| *High* | 132 (42.8) | 3,591 (38.9) | 0.90 (0.59 to 1.39) | 1.06 (1.01 to 1.12) | 0.75 (0.46 to 1.20) |
| *% is column % except for age which is row % of the total | | | | | |


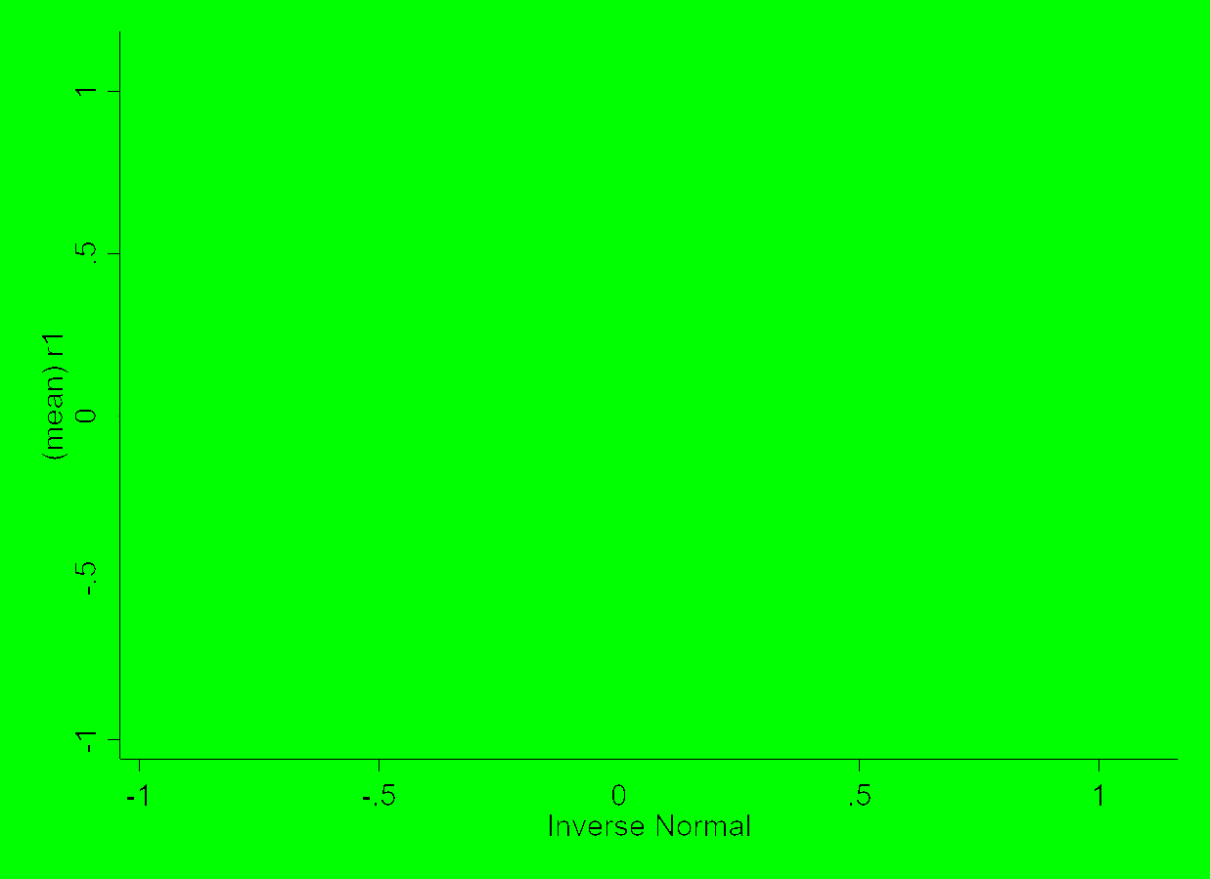


Quantile to quantile plot of the fully adjusted model to determine if well fit


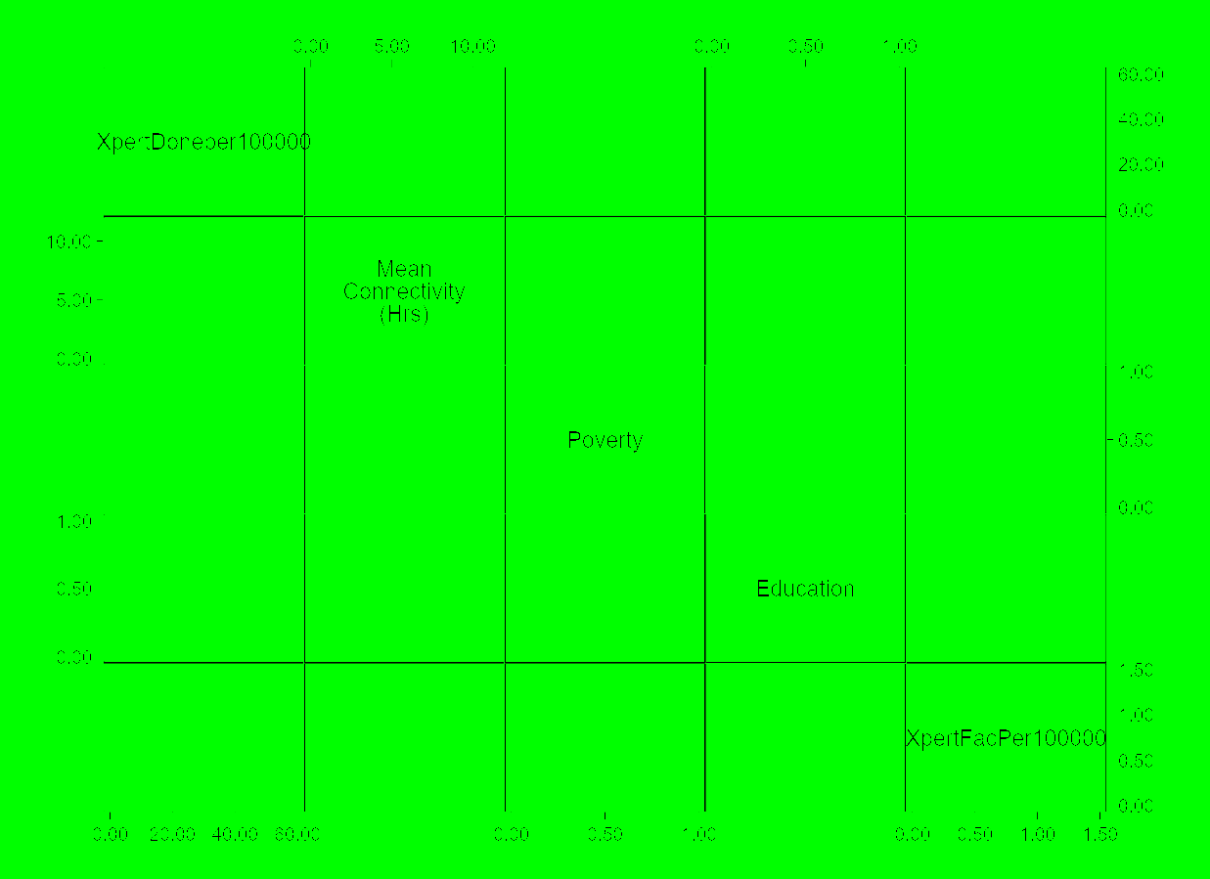


Testing for collinearity amongst continuous variables
