# Supplementary material for: Variability in distribution and use of tuberculosis diagnostic tests in Kenya: a cross-sectional survey
Source: BMC Infect Dis. 2018 Jul 16;18:328. doi: 10.1186/s12879-018-3237-z (PMC6048895; doi:10.1186/s12879-018-3237-z)
Supplement: Supplementary file 3 — Distribution of TB cases by age group for the various counties. (DOCX 27 kb) [file 12879_2018_3237_MOESM3_ESM.docx]

## Additional File 2: Distribution of TB cases by age group for the various counties

| **County** | **Total TB Cases** | **Proportion of Total TB cases** | **Cases in 0-14yrs** | **0-14yrs %**  **of county cases** | **Cases in ≥15yrs** | **≥15yrs % of county cases** | **2015 County Population Projection** | **2015 County CNR/100, 000** |
| --- | --- | --- | --- | --- | --- | --- | --- | --- |
| Baringo | 616 | 0.75 | 48 | 7.79 | 568 | 92.21 | 661,148 | 93.17 |
| Bomet | 1,466 | 1.78 | 121 | 8.25 | 1,345 | 91.75 | 846,012 | 173.28 |
| Bungoma | 1,519 | 1.85 | 157 | 10.34 | 1,362 | 89.66 | 1,655,281 | 91.77 |
| Busia | 1,196 | 1.45 | 106 | 8.86 | 1,090 | 91.14 | 896,025 | 133.48 |
| Elgeyo Marakwet | 510 | 0.62 | 50 | 9.80 | 460 | 90.20 | 435,906 | 117.00 |
| Embu | 1,487 | 1.81 | 185 | 12.44 | 1,302 | 87.56 | 561,446 | 264.85 |
| Garissa | 844 | 1.03 | 131 | 15.52 | 713 | 84.48 | 785,976 | 107.38 |
| Homa Bay | 2,172 | 2.64 | 156 | 7.18 | 2,016 | 92.82 | 1,119,769 | 193.97 |
| Isiolo | 544 | 0.66 | 77 | 14.15 | 467 | 85.85 | 178,199 | 305.28 |
| Kajiado | 1,608 | 1.95 | 159 | 9.89 | 1,449 | 90.11 | 898,289 | 179.01 |
| Kakamega | 2,208 | 2.68 | 198 | 8.97 | 2,010 | 91.03 | 1,929,401 | 114.44 |
| Kericho | 1,707 | 2.07 | 128 | 7.50 | 1,579 | 92.50 | 881,064 | 193.74 |
| Kiambu | 3,709 | 4.51 | 204 | 5.50 | 3,505 | 94.50 | 1,921,392 | 193.04 |
| Kilifi | 1,921 | 2.33 | 177 | 9.21 | 1,744 | 90.79 | 1,336,590 | 143.72 |
| Kirinyaga | 1,376 | 1.67 | 96 | 6.98 | 1,280 | 93.02 | 577,783 | 238.15 |
| Kisii | 1,673 | 2.03 | 104 | 6.22 | 1,569 | 93.78 | 1,306,652 | 128.04 |
| Kisumu | 2,963 | 3.60 | 230 | 7.76 | 2,733 | 92.24 | 1,098,560 | 269.72 |
| Kitui | 2,029 | 2.46 | 111 | 5.47 | 1,918 | 94.53 | 1,086,598 | 186.73 |
| Kwale | 1,161 | 1.41 | 127 | 10.94 | 1,034 | 89.06 | 783,261 | 148.23 |
| Laikipia | 732 | 0.89 | 50 | 6.83 | 682 | 93.17 | 457,514 | 160.00 |
| Lamu | 212 | 0.26 | 16 | 7.55 | 196 | 92.45 | 124,092 | 170.84 |
| Machakos | 2,235 | 2.72 | 113 | 5.06 | 2,122 | 94.94 | 1,238,649 | 180.44 |
| Makueni | 1,644 | 2.00 | 82 | 4.99 | 1,562 | 95.01 | 961,738 | 170.94 |
| Mandera | 624 | 0.76 | 73 | 11.70 | 551 | 88.30 | 1,294,917 | 48.19 |
| Marsabit | 647 | 0.79 | 57 | 8.81 | 590 | 91.19 | 343,399 | 188.41 |
| Meru | 3,433 | 4.17 | 225 | 6.55 | 3,208 | 93.45 | 1,536,422 | 223.44 |
| Migori | 1,938 | 2.35 | 122 | 6.30 | 1,816 | 93.70 | 1,152,165 | 168.21 |
| Mombasa | 4,261 | 5.18 | 292 | 6.85 | 3,969 | 93.15 | 1,179,929 | 361.12 |
| Murang'a | 1,918 | 2.33 | 83 | 4.33 | 1,835 | 95.67 | 958,969 | 200.01 |
| Nairobi | 12,536 | 15.23 | 753 | 6.01 | 11,783 | 93.99 | 3,942,054 | 318.01 |
| Nakuru | 3,695 | 4.49 | 233 | 6.31 | 3,462 | 93.69 | 1,925,296 | 191.92 |
| Nandi | 724 | 0.88 | 54 | 7.46 | 670 | 92.54 | 906,881 | 79.83 |
| Narok | 1,631 | 1.98 | 167 | 10.24 | 1,464 | 89.76 | 1,002,968 | 162.62 |
| Nyamira | 733 | 0.89 | 41 | 5.59 | 692 | 94.41 | 667,716 | 109.78 |
| Nyandarua | 747 | 0.91 | 44 | 5.89 | 703 | 94.11 | 688,618 | 108.48 |
| Nyeri | 1,369 | 1.66 | 56 | 4.09 | 1,313 | 95.91 | 713,823 | 191.78 |
| West Pokot | 1,394 | 1.69 | 235 | 16.86 | 1,159 | 83.14 | 700,414 | 199.03 |
| Samburu | 590 | 0.72 | 66 | 11.19 | 524 | 88.81 | 292,484 | 201.72 |
| Siaya | 1,959 | 2.38 | 137 | 6.99 | 1,822 | 93.01 | 932,108 | 210.17 |
| Taita Taveta | 536 | 0.65 | 59 | 11.01 | 477 | 88.99 | 329,383 | 162.73 |
| Tana River | 424 | 0.52 | 52 | 12.26 | 372 | 87.74 | 284,505 | 149.03 |
| Tharaka Nithi | 992 | 1.21 | 108 | 10.89 | 884 | 89.11 | 406,787 | 243.86 |
| Trans Nzoia | 1,355 | 1.65 | 166 | 12.25 | 1,189 | 87.75 | 1,022,277 | 132.55 |
| Turkana | 2,245 | 2.73 | 385 | 17.15 | 1,860 | 82.85 | 1,256,152 | 178.72 |
| Uasin Gishu | 1,667 | 2.03 | 109 | 6.54 | 1,558 | 93.46 | 1,123,165 | 148.42 |
| Vihiga | 824 | 1.00 | 55 | 6.67 | 769 | 93.33 | 591,138 | 139.39 |
| Wajir | 539 | 0.65 | 52 | 9.65 | 487 | 90.35 | 800,576 | 67.33 |
|  |  |  |  |  |  |  |  |  |
| **Total** | **82,313** | **100.00** | **6,450** | **8.70** | **75,863** | **91.30** | **45,793,491** | **173.23** |
