# Supplementary material for: Variability in distribution and use of tuberculosis diagnostic tests in Kenya: a cross-sectional survey
Source: BMC Infect Dis. 2018 Jul 16;18:328. doi: 10.1186/s12879-018-3237-z (PMC6048895; doi:10.1186/s12879-018-3237-z)
Supplement: Supplementary file 4 — TB Diagnostic tests by county per 100,000 total population. (DOCX 23 kb) [file 12879_2018_3237_MOESM4_ESM.docx]

## Additional File 3: County Names, Case Notification Rates and use of Xpert® and Microscopy

| **County** | **County Name in Full** | **CNR/ 100,000 (0-14yrs)** | **CNR/**  **100,000 (≥15yrs)** | **Xpert/**  **100,000 (0-14yrs)** | **Xpert/**  **100,000 (≥15yrs)** | **Microscopy /100,000 (0-14yrs)** | **Microscopy /100,000 (≥15yrs)** |
| --- | --- | --- | --- | --- | --- | --- | --- |
| Nan | Nandi | 13 | 132 | 0.7 | 26.2 | 3.6 | 97.0 |
| Nya | Nyamira | 12 | 160 | 0.6 | 13.9 | 8.5 | 142.7 |
| Nya | Nyandarua | 14 | 164 | 0.6 | 14.5 | 4.4 | 133.5 |
| Bun | Bungoma | 21 | 168 | 0.9 | 21.0 | 5.6 | 131.9 |
| Bar | Baringo | 16 | 175 | 0.3 | 4.9 | 5.0 | 142.4 |
| Vih | Vihiga | 16 | 181 | 1.2 | 29.5 | 7.0 | 135.0 |
| Kak | Kakamega | 21 | 187 | 1.5 | 28.2 | 7.9 | 146.9 |
| Elg | Elgeyo Marakwet | 25 | 190 | 1.0 | 28.2 | 4.4 | 150.3 |
| Kis | Kisii | 16 | 193 | 2.4 | 37.2 | 7.2 | 177.8 |
| Nye | Nyeri | 16 | 194 | 1.5 | 40.5 | 2.6 | 155.6 |
| Tai | Taita Taveta | 41 | 197 | 2.1 | 33.5 | 7.6 | 161.4 |
| Waj | Wajir | 21 | 203 | 0.4 | 8.3 | 10.3 | 168.0 |
| Mur | Murang'a | 16 | 206 | 2.9 | 39.7 | 6.9 | 184.5 |
| Gar | Garissa | 44 | 223 | 0.3 | 5.3 | 8.1 | 169.8 |
| Mak | Makueni | 16 | 232 | 1.1 | 27.3 | 5.7 | 194.9 |
| Lai | Laikipia | 24 | 239 | 1.5 | 42.8 | 5.8 | 206.8 |
| Bus | Busia | 25 | 243 | 1.4 | 29.9 | 7.9 | 188.3 |
| Mac | Machakos | 20 | 246 | 0.7 | 22.1 | 4.9 | 200.5 |
| Tra | Trans Nzoia | 40 | 252 | 1.0 | 27.9 | 8.9 | 208.3 |
| Kwa | Kwale | 35 | 254 | 1.1 | 25.3 | 6.6 | 193.4 |
| Kil | Kilifi | 30 | 260 | 1.7 | 37.7 | 8.2 | 226.0 |
| Kir | Kirinyaga | 41 | 265 | 2.1 | 62.1 | 5.1 | 222.0 |
| Tan | Tana River | 36 | 267 | 0.7 | 25.1 | 7.0 | 217.7 |
| Uas | Uasin Gishu | 26 | 267 | 1.0 | 27.3 | 6.5 | 203.7 |
| Bom | Bomet | 30 | 275 | 0.2 | 7.0 | 7.3 | 205.7 |
| Kit | Kitui | 19 | 283 | 1.5 | 31.4 | 6.6 | 237.9 |
| Kia | Kiambu | 31 | 285 | 1.5 | 20.0 | 8.3 | 232.9 |
| Emb | Embu | 71 | 296 | 0.0 | 27.0 | 8.8 | 231.1 |
| Lam | Lamu | 34 | 299 | 2.2 | 50.4 | 19.4 | 270.4 |
| Ker | Kericho | 32 | 303 | 1.8 | 42.0 | 12.6 | 254.3 |
| Man | Mandera | 36 | 305 | 0.0 | 8.3 | 15.1 | 249.3 |
| Sia | Siaya | 28 | 305 | 2.1 | 59.9 | 8.2 | 232.9 |
| Nak | Nakuru | 29 | 308 | 0.8 | 30.1 | 6.9 | 258.4 |
| Mer | Meru | 33 | 309 | 0.9 | 41.9 | 7.1 | 249.6 |
| Tha | Tharaka Nithi | 54 | 320 | 1.5 | 31.8 | 9.4 | 232.9 |
| Hom | Homa Bay | 28 | 338 | 2.2 | 63.9 | 12.0 | 266.1 |
| Nar | Narok | 40 | 347 | 2.4 | 48.7 | 15.3 | 283.9 |
| Mig | Migori | 24 | 350 | 0.4 | 16.8 | 8.3 | 295.8 |
| Kis | Kisumu | 41 | 373 | 5.5 | 84.2 | 12.8 | 278.9 |
| Kaj | Kajiado | 63 | 376 | 2.0 | 32.4 | 15.1 | 331.7 |
| Mar | Marsabit | 43 | 389 | 0.7 | 13.8 | 24.7 | 344.5 |
| Nai | Nairobi | 60 | 403 | 3.1 | 45.1 | 16.1 | 289.7 |
| Tur | Turkana | 118 | 478 | 2.8 | 19.5 | 52.3 | 425.7 |
| Sam | Samburu | 60 | 482 | 3.6 | 68.0 | 16.3 | 423.9 |
| Pok | Pokot | 94 | 503 | 1.6 | 55.5 | 23.6 | 414.3 |
| Mom | Mombasa | 77 | 515 | 2.9 | 56.3 | 17.9 | 414.1 |
| Isi | Isiolo | 116 | 558 | 9.1 | 75.3 | 33.2 | 460.3 |
|  |  |  |  |  |  |  |  |
| Mean |  | 34 | 287 | 1.6 | 34.9 | 9.8 | 230.3 |
