# Supplementary material for: Variability in distribution and use of tuberculosis diagnostic tests in Kenya: a cross-sectional survey
Source: BMC Infect Dis. 2018 Jul 16;18:328. doi: 10.1186/s12879-018-3237-z (PMC6048895; doi:10.1186/s12879-018-3237-z)
Supplement: Supplementary file 5 — County Names, Case Notification Rates and use of Xpert® and Microscopy. (DOCX 21 kb) [file 12879_2018_3237_MOESM5_ESM.docx]

## Additional File 4: Distribution of TB Diagnostic tests by county per 100,000 total population

| **County** | **Microscopy Facilities** | **Microscopy Per 100,000** | **Xpert Facilities** | **XPert Per 100,000** | **X-ray Facilities** | **X-ray Per 100,000** |
| --- | --- | --- | --- | --- | --- | --- |
| Baringo | 34 | 5.45 | 2 | 0.32 | 3 | 0.48 |
| Bomet | 21 | 2.34 | 2 | 0.22 | 1 | 0.11 |
| Bungoma | 90 | 5.76 | 2 | 0.13 | 5 | 0.32 |
| Busia | 47 | 5.43 | 2 | 0.23 | 10 | 1.16 |
| Elgeyo Marakwet | 24 | 5.39 | 1 | 0.22 | 2 | 0.45 |
| Embu | 55 | 7.85 | 2 | 0.29 | 5 | 0.71 |
| Garissa | 20 | 3.25 | 3 | 0.49 | 1 | 0.16 |
| Homa Bay | 82 | 7.16 | 4 | 0.35 | 5 | 0.44 |
| Isiolo | 5 | 3.34 | 2 | 1.33 | 1 | 0.67 |
| Kajiado | 36 | 5.65 | 2 | 0.31 | 3 | 0.47 |
| Kakamega | 73 | 3.63 | 1 | 0.05 | 6 | 0.30 |
| Kericho | 44 | 4.80 | 3 | 0.33 | 8 | 0.87 |
| Kiambu | 78 | 4.15 | 5 | 0.27 | 19 | 1.01 |
| Kilifi |  | 0.00 | 3 | 0.24 | 6 | 0.48 |
| Kirinyaga | 47 | 6.53 | 2 | 0.28 | 2 | 0.28 |
| Kisii | 75 | 5.08 | 2 | 0.14 | 7 | 0.47 |
| Kisumu | 88 | 6.81 | 8 | 0.62 | 10 | 0.77 |
| Kitui | 57 | 4.48 | 3 | 0.24 | 4 | 0.31 |
| Kwale | 37 | 4.81 | 3 | 0.39 | 2 | 0.26 |
| Laikipia | 24 | 4.90 | 2 | 0.41 | 5 | 1.02 |
| Lamu | 10 | 8.94 | 1 | 0.89 | 2 | 1.79 |
| Machakos | 55 | 3.89 | 3 | 0.21 | 7 | 0.49 |
| Makueni | 46 | 3.85 | 3 | 0.25 | 3 | 0.25 |
| Mandera | 16 | 4.15 | 1 | 0.26 | 2 | 0.52 |
| Marsabit | 12 | 4.21 | 3 | 1.05 | 16 | 5.61 |
| Meru | 2 | 0.12 | 5 | 0.29 | 9 | 0.53 |
| Migori | 72 | 7.03 | 3 | 0.29 | 6 | 0.59 |
| Mombasa | 51 | 4.43 | 7 | 0.61 | 13 | 1.13 |
| Muranga | 61 | 4.32 | 2 | 0.14 | 6 | 0.42 |
| Nairobi | 159 | 3.82 | 14 | 0.34 | 42 | 1.01 |
| Nakuru | 80 | 4.16 | 2 | 0.10 | 9 | 0.47 |
| Nandi | 33 | 3.59 | 2 | 0.22 | 6 | 0.65 |
| Narok | 42 | 5.00 | 2 | 0.24 | 34 | 4.05 |
| Nyamira | 62 | 8.02 | 2 | 0.26 | 1 | 0.13 |
| Nyandarua | 41 | 5.49 | 1 | 0.13 | 4 | 0.54 |
| Nyeri | 3 | 0.29 | 2 | 0.20 | 9 | 0.88 |
| Pokot | 27 | 5.62 | 2 | 0.42 | 1 | 0.21 |
| Samburu | 5 | 2.28 | 1 | 0.46 | 2 | 0.91 |
| Siaya | 77 | 7.10 | 3 | 0.28 | 3 | 0.28 |
| Taita Taveta | 42 | 10.88 | 2 | 0.52 | 3 | 0.78 |
| Tana River | 21 | 7.44 | 1 | 0.35 |  | 0.00 |
| Tharaka Nithi | 26 | 5.44 | 2 | 0.42 | 3 | 0.63 |
| Trans Nzoia |  | 0.00 | 1 | 0.11 | 4 | 0.45 |
| Turkana | 28 | 3.92 | 3 | 0.42 | 3 | 0.42 |
| Uasin Gishu | 33 | 3.31 | 3 | 0.30 | 8 | 0.80 |
| Vihiga | 32 | 4.17 | 2 | 0.26 | 2 | 0.26 |
| Wajir | 21 | 4.26 | 1 | 0.20 | 1 | 0.20 |
